# Evaluation of Platelet-Rich Plasma by means of PRGF^®^-Endoret^®^ protocol in leukemia cats: PDGF-BB and TGF-ß1 valuation

**DOI:** 10.3389/fvets.2023.1110055

**Published:** 2023-01-26

**Authors:** Laura Miguel-Pastor, Katy Satué, Deborah Chicharro, Pau Peláez, Marta Torres-Torrillas, José M. Carrillo, José J. Cerón, Joaquín J. Sopena, Mónica Rubio

**Affiliations:** ^1^Bioregenerative Medicine and Applied Surgery Research Group, Department of Animal Medicine and Surgery, CEU Cardenal Herrera University, CEU Universities, Valencia, Spain; ^2^García Cugat Foundation CEU-UCH Chair of Medicine and Regenerative Surgery, CEU Cardenal Herrera University, CEU Universities, Valencia, Spain; ^3^Interdisciplinary Laboratory of Clinical Analysis, University of Murcia, Murcia, Spain

**Keywords:** plasma rich in growth factors, PRGF^®^-Endoret^®^, feline leukemia, FeLV, cat, PDGF-BB, TGF-ß1, platelet

## Abstract

**Introduction:**

Feline leukemia virus (FeLV) is a chronic disease that leads to the weakening of a cat's immune system. Platelet-rich plasma (PRP) offers therapeutic effects for multiple diseases, the use of PRP and growth factors (GFs) determination could be an alternative treatment to improve the quality of life in these patients. The objectives of this study were to determine and compare the concentration of platelets (PLTs), red blood cells (RBCs) and white blood cells (WBCs) between samples of whole blood (WB), PRP and platelet-poor plasma (PPP) fractions, and to evaluate the concentration of platelet-derived growth factor BB (PDGF-BB) and transforming growth factor β1 (TGF-β1) in both fractions in FeLV cats using a PRGF^®^-Endoret^®^ protocol previously standardized in this species.

**Methods:**

WB was collected from 11 asymptomatic FeLV-positive cats. PRP and PPP was obtained following PRGF^®^-Endoret^®^ technology according to centrifugation at 265 g for 10 min. Cellular components, RBCs, WBCs, PLTs, and the PDGF-BB and TGF-β1 concentrations in PRP and PPP fractions were determined.

**Results:**

PLT in the PRP fraction was statistically higher than WB and PPP fraction, with no statistical differences between WB and PPP. PLT concentration increased 1.4 times in PRP fraction compared to WB. Mean platelet volume (MPV) did not differ significantly between the WB, PRP, and PPP fractions. Compared to WB, the absolute numbers of RBCs and WBCs were decreased by 99% and more than 95% in the PRP and PPP fractions, respectively. TGF-ß1 concentrations increased in PRP vs. PPP, with no changes in PDGF-BB.

**Discussion:**

Based on the degree of PLT enrichment and the absence of RBCs and WBCs, this blood product could be classified as a Pure Platelet-Rich Plasma (P-PRP). The presence of GFs in PRP and PPP samples suggests that the PRGF^®^-Endoret^®^ methodology is suitable for obtaining PRP in FeLV cats, despite future studies are necessary to optimize the technique, standardize the results and assess clinical efficacy.

## 1. Introduction

Platelet-rich plasma (PRP) is an autologous blood product defined by a platelet (PLT) concentration higher than baseline. Inside, the PLTs have granules that, when activated, release growth factors (GFs), cytokines, and chemokines into the medium. Some of the most important GFs released by PLTs in PRP include platelet-derived growth factor (PDGF, A-B-C), transforming growth factor beta1 (TGF-ß1), vascular endothelial growth factor (VEGF), fibroblast growth factor (FGF), hepatocyte growth factor (HGF), connective tissue growth factor (CTGF), epidermal growth factor (EGF), and insulin-like growth factor-1 (IGF-1), which are involved in tissue restoration and immunomodulation (1–4).

Taking into account the cellular content and the presence or absence of fibrin, four main groups of PRP products have been identified (5): Pure Platelet-Rich Plasma (P-PRP), Leukocyte and Platelet-Rich Plasma (LR-PRP), Pure Platelet-Rich Fibrin (P-PRF), and Leukocyte and Platelet-Rich Fibrin (L-PRF). P-PRP, as a PRGF^®^-Endoret^®^ contains a moderate PLT concentration and no leukocytes (WBCs); LR-PRP includes a WBC fraction, but the fibrin matrix is sparse; P-PRF is a blood product with very low concentration of WBCs and is collected owing to the specific separator gel used in the method, PLT activation and fibrin polymerization are triggered using calcium chloride. L-PRF is considered as second-generation PLT concentrate because the natural concentrate is produced without any anticoagulants or gelifying agents, and after centrifugation the PRF clot forms a strong fibrin matrix in which most of the PLTs and WBCs from the harvested blood are concentrated. Continuous revisions of terminology and new classification criteria for PRP bioformulations in regenerative medicine (2) have made it possible to identify up to 15 types of PRP products with differences in formulation, biological properties and mechanisms of action, as well as different clinical indications and clinical results (6, 7). The topical use of PLT concentrates is recent, and its efficiency remains controversial. Several techniques for PLT concentrates are available, and their applications have been confusing because each method leads to a different product with a different biology and potential uses (8). Even though PRP has been widely used in veterinary medicine, no valid standardized classification methods for platelet-derived products have been currently published.

Plasma-Rich Growth Factors (PRGF) represents a novel technology that uses autologous proteins and GFs derived from PLTs as therapeutic formulations for regenerative purposes, cell repair and regeneration. PRGF^®^-Endoret^®^ technology following a unique centrifugation protocol allows a PRP product to be obtained with moderate PLT enrichment and an extreme reduction of WBCs and RBCs, which decreases the proinflammatory activity of WBCs. The PRGF^®^-Endoret^®^ protocol for obtaining GFs is widely documented in different species and has been successfully applied in different clinical areas (9–11), such as regenerative medicine for wound healing, ophthalmology, dentistry, osteoarthritis, tendinopathies, or aesthetic medicine in humans (12–19); traumatology and ophthalmology in dogs (20–22) and horses (23); and wound healing (24, 25) or the treatment of complete cartilage defects in rabbits (26). Although different protocols for obtaining PRP in cats have been described (27–32), the use of PRGF^®^-Endoret^®^ technology in this species has only been reported in a previous study carried out by the same researchers. Due to the cellular characteristics of the autologous blood product obtained following centrifugation, it was classified as a P-PRP product (33). Despite this, there is no clinical evidence of its application, and therefore future research is needed in this field of feline internal medicine (28, 29, 33).

Feline viral leukemia is caused by feline leukemia virus (FeLV), one of the retroviruses with the greatest impact on feline health worldwide. The prevalence is highly variable depending on the geographic location and the animal population analyzed, varying between 3.0 and 28.4% in South America, 0.5 and 24.5% in Asia and Australia/New Zealand, 2.3 and 3.3% in the USA, and 0.7 and 15.6% in Europe, among others (34–40). In Spain, specifically, the reported prevalence of FeLV is 2.6% (41).

Following the course of the infection, four different forms of FeLV infection have been identified: abortive infection (“regressor cats”), regressive infection (“transient viremia” followed by “latent infection”), progressive infection (“persistent viremia”), and focal or atypical infection (42). Progressive infections are categorized by detectable antigenemia, FeLV RNA, and DNA provirus, since the virus persistently sheds into the circulation and tissues have low or no antibodies to FeLV (42). In 30–40% of cats with progressive infection, FeLV virus has tropism for the hematopoietic cells in the bone marrow (BM) associated with proliferative, degenerative, and oncogenic diseases in erythroid, myeloid, and lymphoid cell lineages (42). Both lymphoma (43) and myelodysplastic syndrome are relatively common abnormalities in FeLV-infected cats. Non-neoplastic diseases, such as regenerative anemia, immunosuppression, neutropenia, lymphopenia, and PLT abnormalities like thrombocytopenia or PLT function alterations, can occur in animals persistently infected with FeLV (35, 44–46). Moreover, ~80% of cats die before 4–5 years of life; prognosis for cats with progressive infections is variable depending upon current immune status, stress, or concurrent disease such that cats can remain without clinical signs for several years after the infection of the BM (47, 48).

Due to the fact that GFs promote wound healing or tissue regeneration in musculoskeletal injuries, such as osteoarthritis in dogs (20–22), horses (23), rabbits (26), and humans (49, 50), the authors considered that GFs could be an advantageous therapeutic option in cats with FeLV. For this reason, the objective of the study was to determine PLT concentrations and two specific types of GFs, such as Platelet-Derived Growth Factor-BB (PDGF-BB) and Transforming Growth Factor-ß1 (TGF-ß1), in both PPP and PRP fractions following the PRGF^®^-Endoret^®^ protocol previously standardized for its application in cats by these same researchers (33). The authors hypothesize that a P-PRP can be obtained in FeLV cats following PRGF^®^-Endoret^®^ methodology as in other species or in healthy cats.

## 2. Material and methods

### 2.1. Animals

The study protocol was approved by the Animal Welfare Ethics Committee (CEEA) of the CEU Cardenal Herrera University in Valencia (Spain) in accordance with the Spanish Animal Protection Policy (RD53/2013), which complies with the European Union Directive European 2010/63/EU, with the following approval code: 2018/VSC/PEA/0196.

This prospective study included cats brought to CEU-Cardenal Herrera University Veterinary Clinical Hospital between October 2021 and July 2022 as part of a medical clinical study, during which cats were examined for retroviral infections. A total of fourteen non-pedigree FeLV-positive adult cats were included in the study. All animal owners agreed to participate in the study by signing a consent form.

Only animals clinically healthy and with two positive subsequent test results for FeLV by the commercially available combined enzyme-linked immunosorbent assay (ELISA) kit for feline immunodeficiency virus (FIV) antibody and antigen FeLV p27 (IDEXX SNAP^®^ Combo FeLV Ag/FIV Antibody Test) were included in the study. The first samples with positive results were subsequently tested again from 6 to 9 months after, following the same methodology, considering true positives or progressive infection by FeLV those that tested positive twice. Discordant test results were considered negative.

Animals receiving any treatment for the previous or current 6 months of the study and those that developed diseases or tested positive for both FIV and FeLV or only FIV were excluded from the study. Animals with hematological alterations consisting of anemia, leukopenia or true thrombocytopenia were also excluded. Each patient was monitored by a veterinarian throughout the entire procedure.

### 2.2. Sample processing

To reduce stress, cats were first intramuscularly (IM) sedated with a combination of butorphanol (0.3 mg/kg), dexmedotomidine (12 μg/kg), and alfaxalone (0.8 mg/kg). Following sedation, the cephalic vein was catheterized using a 22 G catheter to collect 0.5 ml of blood, which was immediately transferred to a 0.5 ml tube containing K3-EDTA (BD Vacutainer; Becton, Dickinson) for blood count or whole blood (WB) analysis. Thereafter, 9 ml of blood was collected in sterile conditions from the external jugular vein by means of a vacutainer sodium citrate 3.8% tube (Blood-Collecting Tubes^®^, BTI Biotechnology Institute, Alava, Spain) for PRGF^®^-Endoret^®^ preparation. Subsequently, each cat received 9 ml of acetated Ringer's solution IV during the first 20 min to restore the vascular volume and to prevent hemodynamic complications.

### 2.3. PRGF^®^-Endoret^®^ processing

Feline samples collected in sodium citrate tubes were immediately centrifugated at room temperature in a PRGF^®^-Endoret^®^ System IV centrifuge (BTI Biotechnology Institute S.L.) under a single centrifugation protocol of 265 g for 10 min as described by Miguel-Pastor et al. (33).

**Figure 1:**
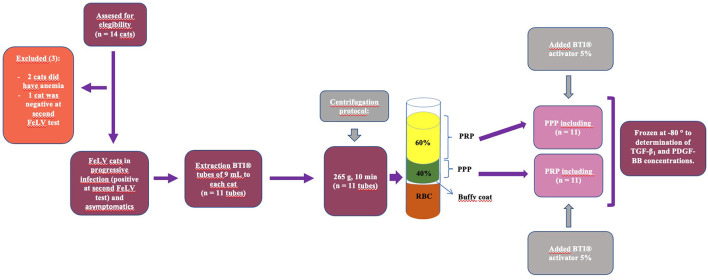
Schematization of the study design: blood sample obtention, centrifugation following feline BTI protocol using the PRGF^®^-Endoret^®^ methodology, PRP and PPP fraction obtention, subsequent activation of both fractions with BTI-activator and freezing at −80°C for subsequent GFs analysis.

Following the PRGF^®^-Endoret^®^ methodology, two fractions were obtained after centrifugation: platelet-poor plasma (PPP) and PRP. Sixty percentage of the upper plasma was considered PPP, and the remaining 40% above the “buffy coat” was considered the PRP fraction. Both fractions were pipetted under maximum sterile conditions with a laminar flow hood and always by the same researcher. Subsequently, samples were transferred to individual fractionation tubes with no additives (PRGF^®^ fractionation tubes, BTI, Institute of Biotechnology, Álava, Spain). In addition, the plasma fractions (PPP and PRP) were activated by adding 5% of the plasma volume of 10% calcium gluconate (activator PRGF^®^, Institute of Biotechnology, Álava, Spain) to achieve PLT degranulation and release of the GFs, obtaining PRGF. Plasma samples were then aliquoted into eppendorf tubes and immediately frozen at −80°C following PLT activation for subsequent determination of TGF-ß1 and PDGF-BB concentrations (Figure 1).

### 2.4. Hematological analysis

A complete automated blood count was performed for each feline specimen using the Advia^®^ 2120i (Advia^®^ 2120i Siemens Healthcare Diagnostics Inc.), including red blood cell (RBC; 10e^6^/μL), hemoglobin concentration (HB; g/dL), packed cell volume (PCV; %), mean corpuscular volume (MCV; fL), mean corpuscular hemoglobin (MCH; pg), mean corpuscular hemoglobin concentration (MCHC; g/dL), mean hemoglobin content (CH; pg), hemoglobin concentration distribution width (HDW; d/dL), red blood cell distribution width (RDW; %), reticulocytes absolute count (RET; 10e^9^/L), white blood cell (WBC; 10e^3^/μL), neutrophils (NFS; 10e^3^/μL), lymphocytes (LYMPH; 10e^3^/μL), monocytes (MON; 10e^3^/μL), eosinophils (EOS; 10e^3^/μL), and basophils (BAS; 10e^3^/μL). Platelet (PLT; K/μL) counts and mean platelet volume (MPV; fL) were also determined in WB samples. In the PRP and PPP fractions, RBC, PLT, WBC, LYMPH, NFS, and MON counts, as well as the MPV, were also determined. Verification of absolute PLT numbers, such as the presence of PLT aggregates and differential WBC counts obtained by ADVIA, was performed on Romanowsky-stained blood smears and always by the same pathologist.

### 2.5. Platelet-Derived Growth Factor-BB and Transforming Growth Factor-ß1 quantification

The concentrations of both GFs in both plasma fractions (PPP and PRP) were determined using an ELISA kit of development with antibodies to human (Human TGF-beta1 DuoSet ELISA de R&D Systems DY240-05 and Human PDGF-BB DuoSet ELISA de R&D Systems DY220, respectively), following the methodology previously published by Miguel-Pastor et al. (33). To the knowledge of the authors, there are no commercial kits for GF determination in cats, so human kits were used for GFs determination as described by other researchers (27, 51).

### 2.6. Statistical methods

Data were analyzed using SPSS 20.0 for Windows (SPSS^®^ Inc., Chicago, USA). A descriptive study of the mean, standard deviation and confidence intervals was performed for each variable. The normality of the data was verified in each variable with the Shapiro–Wilk test, and the homogeneity of the variance was verified with the Levene test. The means of the variables were studied using a linear mixed model. These models included the treatment group as fixed effects and the cat as a random effect. If the interaction was found statistically significant, analyses using a one-way ANOVA and a Bonferroni test were used. Non-parametric Kruskal–Wallis tests were used to compare variables not adjusted to a normal distribution. *P* < 0.05 was considered significant.

## 3. Results

### 3.1. Animals and sample acquisition

Of the 14 cats initially included in the study, three of them were excluded: two presented moderate anemia and one tested negative on the second FeLV test. A total of 11 sterilized cats met the inclusion criteria, six males and five females, aged between 1.5 and 6.5 years (Mean: 4.4; SD: 1.6), and weighing between 3.7 and 5.8 kg (Mean: 4.7; SD: 0.7).

The collection and centrifugation of blood were performed with no intercurrence in all cats. Following this procedure, the three fractions (erythrocyte, buffy coat area, and plasma fraction) were obtained.

Table 1 shows the mean values ± SD or median and the 95% CI of all the hematological parameters considered in WB. Comparisons between RBC, PLT, WBC, LYMPH, NFS, and MON counts, as well as MPV between WB and PRP and PPP fractions, and comparisons between PDGF-BB and TGF-ß1 between PRP and PPP fractions are presented in Table 2. Table 3 shows individually the number of PLTs in WB, PRP, and PPP in relation to the volume of recovered plasma. Table 4 shows the recovered volume of PRP and PPP individually, considering the PCV in each animal.

**Table 1:** Mean ± SD of red blood cell (RBC) count, hemoglobin concentration (HB), packed cell volume (PCV), mean corpuscular volume (MCV), mean corpuscular hemoglobin (MCH), mean corpuscular hemoglobin concentration (MCHC), mean hemoglobin content (CH), hemoglobin concentration distribution width (HDW), red blood cell distribution width (RDW), reticulocytes absolute count (RET), white blood cell (WBC), neutrophil (NFS), lymphocyte (LYMPH), monocyte (MON), eosinophil (EOS), basophil (BAS) and platelet (PLT) counts, and mean platelet volume (MPV) in whole blood samples (WB) in 11 FeLV-positive cats (*n* = 11).

**Parameter**	***N***	**Mean ±SD**	**Minimun**	**Maximum**	**90% CI (lower reference limit)**	**90% CI (upper reference limit)**	**Reference range**
**Erythrogram**							
RBC (10^6^/μl)	11	7.4 ± 1.1	5.9	9.9	6.7	8.1	5.0–10.0
HB (g/dl)	11	10.6 ± 1.6	8.3	14.0	9.5	11.7	8.0–15.0
PCV (%)	11	30.9 ± 4.6	23.0	40.5	27.9	34.1	24.0–45.0
MCV (fl)	11	41.9 ± 3.8	32.2	46.7	39.4	44.4	39.0–55.0
MCH (pg)	11	14.3 ± 1.5	11.2	16.4	13.3	15.3	12.5–17.5
MCHC (g/dl)	11	34.1 ± 1.6	29.7	35.2	33.1	35.2	30.0–36.0
CH (pg)	11	14.8 ± 1.2	11.8	16.1	13.9	15.6	12.0–16.0
HDW (g/dl)	11	2.4 ± 0.2	2.12	2.88	2.2	2.5	1.6–2.9
RDW (%)	11	16.4 ± 0.9	14.6	18.5	15.8	17.1	14.0–18.0
RET (10^9^/μl)	11	54.3 ± 40.6	13.8	134.9	27.1	81.6	15.0–81.0
**Leukogram**							
WBC (10^3^/μl)	11	12.1 ± 5.4	4.31	22.18	8.4	15.7	5.5–19.5
NFS (10^3^/μl)	11	8.7 ± 5.0	2.07	17.9	5.4	12.1	2.5–12.5
LYMPH (10^3^/μl)	11	2.5 ± 0.5	1.9	3.1	2.1	2.8	1.5–7.0
MON (10^3^/μl)	11	0.32 ± 0.19	0.1	0.7	0.2	0.4	0.0–0.9
EOS (10^3^/μl)	11	0.9 ± 1.7	0.0	6.1	0.0	2.0	0.0–0.8
BAS (10^3^/μl)	11	0.3 ± 0.9	0.0	3.0	0.0	0.9	0.0–0.2
**Platelet parameters**							
PLT (10^3^/μl)	11	307.3 ± 106.2	118.0	432.0	235.9	378.6	200–500
MPV (fl)	11	17.03 ± 5.29	10.0	27.7	13.5	20.6	8.6–18.9

Reference values for the cat determined using ADVIA 2021i Laboratory of the Veterinary Clinical Hospital of the CEU-Cardenal Herrera University.

**Table 2:** Mean ± SD of platelet (PLT) concentrations, mean platelet volume (MPV), erythrocytes (RBCs), leukocytes (WBC), lymphocytes (LYMPH), neutrophils (NFS), and monocytes (MON) concentrations in whole blood (WB) samples and in the PRP and PPP fractions; platelet-derived growth factor BB (PDGF-BB) and transforming growth factor B1 (TGF-β1) concentrations in the PRP and PPP fractions in 11 FeLV cats (*n* = 11).

		**Centrifugation protocol (265 g** × **10 min)**	
	**WB**	**PRP**	**PPP**
PLT (10e^3^/μL)	307.3 ± 106.2a	392.1 ± 130.7b	260.8 ± 83.9a
MPV (fL)	17.0 ± 5.3a	14.1 ± 3.6a	13.3 ± 3.4a
RBC (10e^6^/μL)	7.4 ± 1.1a	0.1 ± 0.0b	0.0 ± 0.0b
WBC (10e^3^/μL)	12.1 ± 5.4a	0.6 ± 0.3b	0.3 ± 0.2b
LYMPH (10e^3^/μL)	2.5 ± 0.5a	0.5 ± 0.3b	0.3 ± 0.2b
NFS (10e^3^/μL)	8.7 ± 4.9a	0.1 ± 0.1b	0.0 ± 0.0b
MON (10e^3^/μL)	0.3 ± 0.2a	0.0 ± 0.0b	0.0 ± 0.0b
PDGF-BB (pg/ml)		248.3 ± 152.3a	198.5 ± 143.2a
TGF-β1 (pg/ml)		17,556.9 ± 7,084.2a	11,487.3 ± 3,427.7b

Different letters (a, b) indicate differences between groups. P < 0.05 is considered statistically significant.

**Table 3:** Number of platelets (PLT) in whole blood (WB) and platelet concentrations considering the total volumes of plasma in the PRP and PPP fractions.

**PLT (10e**^**3**^**/**μ**L)**				**% concentration**	
**Animal**	**WB**	**PRP**	**PPP**	**PRP**	**PPP**
1	277	159	123	57.4	44.4
2	421	393	286	93.4	67.9
3	396	295	231	74.5	58.3
4	284	462	364	162.7	128.2
5	118	370	244	313.6	206.8
6	432	670	414	155.1	95.8
7	308	391	301	126.9	97.7
8	375	433	169	115.5	45.1
9	134	273	197	203.7	147.0
10	364	484	295	133.0	81.0
11	271	383	245	141.4	90.4
MEAN	307.3	392.1	260.8	143.4	96.6

**Table 4:** Individual packed cell volume (PCV; %) and recovered volumes of PRP (mL of plasma corresponding to the bottom 40% of the total plasma fraction) and PPP (mL of plasma corresponding to the top 60% of the plasma fraction) for each cat.

**Animal**	**PCV (%) of WB**	**PRP volume (mL)**	**PPP volume (mL)**
1	33.0	2.4	3.6
2	23.9	2.7	4.1
3	36.9	2.3	3.4
4	29.1	2.5	3.8
5	30.4	2.5	3.8
6	29.7	2.5	3.8
7	40.5	2.1	3.2
8	27.7	2.6	3.9
9	29.6	2.5	3.8
10	32.3	2.4	3.7
11	27.8	2.6	3.9

### 3.2. Platelet concentration and mean platelet volume

**Figure 2:**
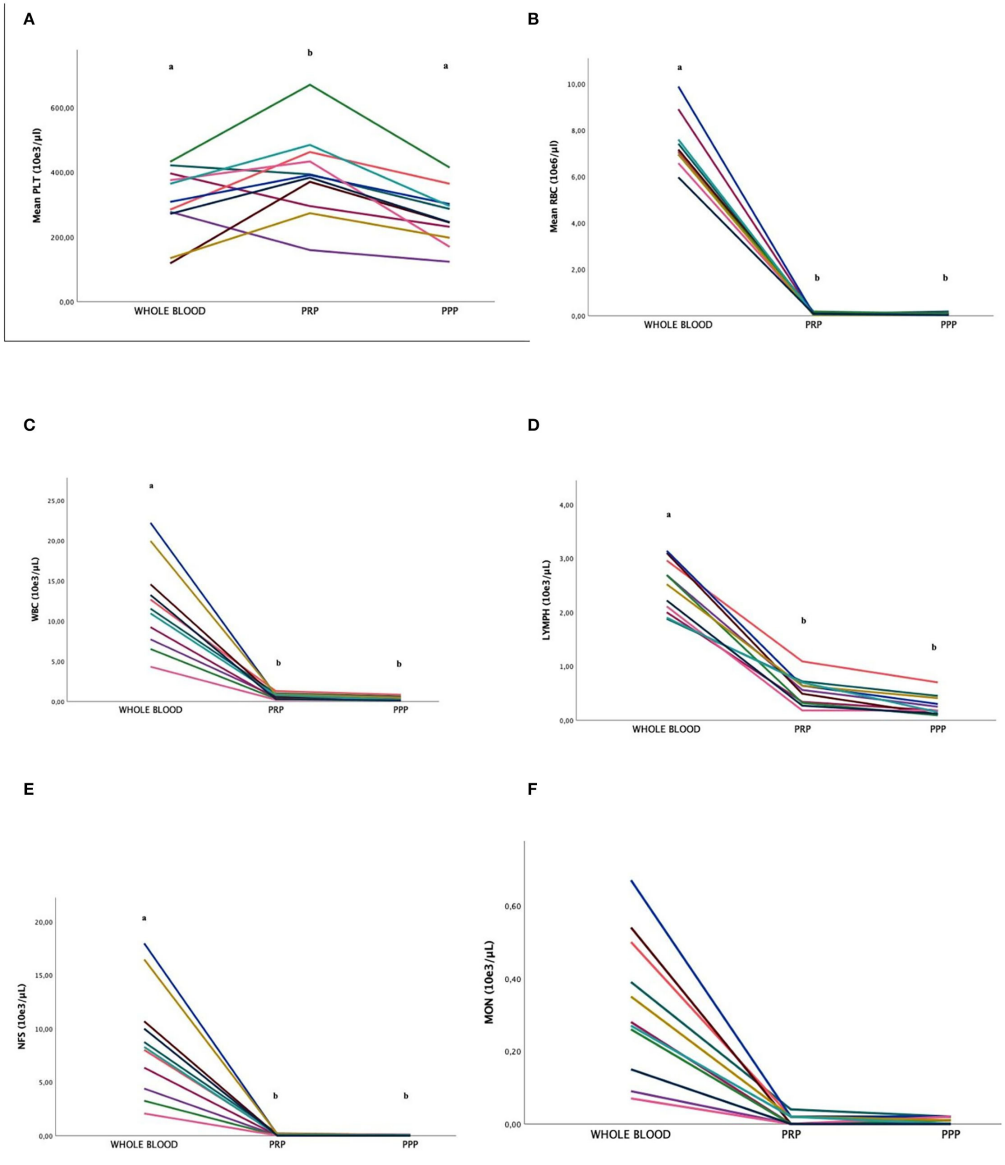
Comparison of the platelet [PLT; **(A)**], red blood cell [RBC; **(B)**], white blood cell [WBC; **(C)**], lymphocytes [LYMPH; **(D)**], neutrophils [NFS; **(E)**], and monocyte [MON; **(F)**] concentrations (mean ± SD) in FeLV cats (*n* = 11) between whole blood (WB) and PRP and PPP fractions. Different letters (a, b) indicate differences between groups. *P* < 0.05 statistically different.

The mean number of PLT was statistically higher in PRP (392.1 ± 130.7 PLTs) compared to PPP (260.8 ± 83.9 PLTs) fraction (*p* = 0.024) and WB (307.3 ± 106.2 PLTs) (*p* = 0.043) with no statistical differences between WB and PPP fraction (Tables 2, 3, Figure 2A). MPV did not differ significantly between the WB, PRP, and PPP fractions.

PLT aggregates were present in 4 (36%) WB samples, 2 (18%) PRP samples, and 2 (18%) PPP samples. According to the equivalence between PLT aggregates by ADVIA 2120i and in smears (51), of the eight samples, seven presented 1–7 aggregates with more than 10 PLTs per field, and the remaining 1–3 aggregates with more than 50 PLTs per field.

### 3.3. RBC concentration

The number of RBCs was statistically higher in WB compared to the PRP and PPP fractions (*p* < 0.01), with no statistical differences between them. The PRGF^®^-Endoret protocol allowed reducing the number of RBCs by 99% in both PRP and PPP fractions (Figure 2B).

### 3.4. WBC concentration

The number of WBCs in WB was significantly higher compared to the PRP and PPP fractions (*p* < 0.01). The mean concentration of WBC was reduced by 95% in the PRP fraction and reduced by 97% in the PPP fraction (Figure 2C).

### 3.5. Lymphocyte concentration

The number of LYMPH was significantly higher in the WB fraction than in the PRP and PPP fractions. LYMPH concentration was significantly decreased by 80% (*p* < 0.01) and 88% in the PRP and PPP fractions (*p* < 0.01), respectively (Figure 2D). The mean number of LYMPH was significantly higher in the WB fraction compared to the PRP and PPP fractions.

### 3.6. Neutrophil concentration

The number of NFS was significantly higher in the WB fraction compared to the PRP and PPP fractions. The mean NFS concentration was significantly decreased by 99% in both PRP and PPP fractions (*p* < 0.01; Figure 2E).

### 3.7. Monocyte concentration

The number of MON was significantly higher in the WB fraction compared to the PRP and PPP fractions. The mean MON concentration was significantly decreased by 97% in both PRP and PPP fractions (*p* < 0.01; Figure 2F).

### 3.8. PDGF-BB and TGF-ß1 concentrations

**Figure 3:**
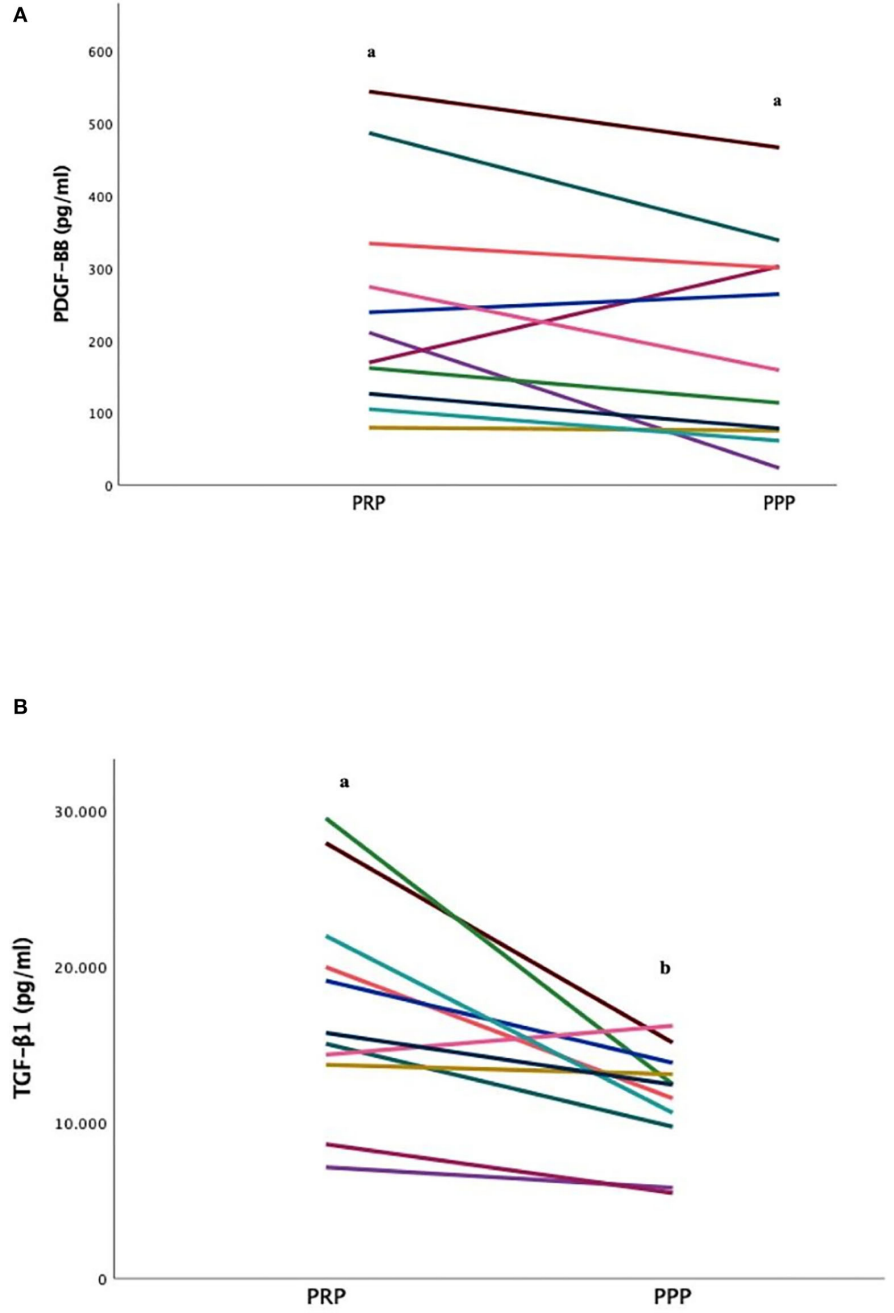
Comparison of Platelet-Derived Growth Factor-BB [PDGF-BB; **(A)**] and transforming growth factor ß1 [TGF-ß1; **(B)**] concentrations (mean ± SD) in FeLV cats (*n* = 11) between PRP and PPP fractions. Different letters (a, b) indicate differences between groups. *P* < 0.05 statistically different.

No significant differences were found between the PRP and PPP fractions in PDGF-BB concentrations (Figure 3A), although in samples with PLT aggregates, the values were higher (*p* = 0.02) than those without aggregates. The mean concentration of TGF-ß1 in the PRP fraction was statistically higher than the PPP fraction (*p* = 0.02) were not affected by the presence of aggregates (Figure 3B).

## 4. Discussion

To the authors' knowledge, this is the first study to separate the PRP and PPP fractions in cats naturally infected with FeLV. The mean PLT enrichment was 1.4 (143%) times higher in the PRP fraction compared to the WB fraction. These results differ little from those observed in a previous study in healthy cats by the same authors in which PLT concentrate was 1.5 (147%) times higher in PRP fraction compared to the WB fraction using the same centrifugation protocol (33). Nevertheless, in other studies in which other types of protocols for obtaining PRP were used, increases in the concentration of PLTs in the PRP fraction were also detected compared to the WB fraction. Thus, Silva et al. showed 183% PLT enrichment in PRP vs. WB using 85 g gravitational force for 6 min in 8.5 mL tubes containing 1.5 mL ACD-A solution (trisodium citrate, citric acid, and dextrose) (27). More recently, Chun et al. reported a 151% PLT enrichment in a 12.5 mL volume of WB in a 30 mL syringe with 2.5 mL citrate and dextrose anticoagulant by double centrifugation at 3,600 rpm for 1 min and then 3,800 rpm for 5 min (29). Likewise, Ferrari and Schwartz (28) evaluated the PLT concentration of PRP within two commercial centrifugation systems. With system 1, 13.5 ml of WB was included in a syringe with 1.5 ml of ACDA and centrifuged at 1,300 rpm for 5 min. Using system 2, 12.5 mL of WB were taken in a syringe with 2.5 mL of ACDA following two consecutive centrifugations at 3,600 rpm for 1 min and at 3,800 rpm for 5 min. While System 1 reduced PLT concentrations by 3%, System 2 increased PLT concentrations by 187% (28). To evaluate the effect of local autologous PRP on healing by secondary intention of skin disorders in cats, a recent study by Angelou et al. (32) included 11 mL of WB in a vacuum tube containing separating gel and anticoagulant (MACD7) and centrifuged at 1,500 rpm for 10 min. PLT counts in the obtained PRP product increased from 2 to 8.2 times compared to WB in this latter study (32). Even though we cannot specify the exact origin of these differences, factors such as WB volume samples, gravitational force, and centrifugation times in the different protocols used to obtain PRP, should be taken into account since the basal PLT number in the WB fraction were similar.

The mean number of PLTs in the PRP fraction in this study was 392.1 ± 130.7 PLTs, not obtaining homogeneous results in all samples, as can be seen in the standard deviation (145.8 PLTs). This was probably the reason why there were no differences found between WB and PPP. In addition, PLT concentrations in PRP of FeLV cats was lower than that obtained previously in healthy animals (481.4 ± 275.0 PLTs) by these same authors (33). A similar trend of PLTs in PPP (260.8 ± 83.9 PLTs) vs. healthy cats (293.3 ± 161.4 PLT) was observed. This fact is probably related to individual variability since in both cases the PRP fraction was obtained using the same sample volume, relative centrifugal force, temperature, and time of centrifugation. The results of this study show that the PPP fraction contains a considerable proportion PLTs that should have been concentrated in the PRP, as a result optimization of the technique is crucial to obtain a more PLT-concentrated PRP. Following the BTI-Endoret^®^ methodology, the PRP fraction was obtained in this study using a single centrifugation protocol. The authors highlight the need of optimizing the protocol with future studies on blood sample centrifugation protocols by varying the gravitational force or the centrifugation time to obtain PRP with a higher concentration of PLTs following PRGF^®^-Endoret^®^ characteristics; or even taking into consideration the need of a second centrifugation to recover more PLTs in the PRP and decrease them in the PPP fractions.

The moderate enrichment in PLT and the absolute reduction in the number of RBCs and WBCs, including LYMPH, NFS, and MON, in both the PRP and PPP fractions enabling a P-PRP in FeLV cats similar to those established in healthy cats by these same researchers (33). However, there was a minimum requirement that a PRP of at least 1.5 should compare to WB according to the PRGF^®^-Endoret^®^ methodology (9) as previously described in healthy cats (33), dogs (20), and rabbits (52). A larger population of sick cats may elucidate the origin of these differences, since none of the animals included in the study presented thrombocytopenia.

Pseudothrombocytopenia in feline blood samples is a common finding on blood smears in both FeLV positive and healthy animals (53). In fact, from the 11 WB samples analyzed, four presented aggregates, or 36%, as previously reported in these same species (33). Other studies have reported higher rates of pseudothrombocytopenia: 40% (28), 57% (54), 62% (53), and 71% (55, 56). The large size of PLTs, the secretion of granules when exposed to high concentrations of serotonin, and irreversible aggregation in response to adenosine diphosphate (ADP) reduction are factors involved in the formation of PLT clumps in these species (57). However, the sampling quality is the main cause of PLT aggregation in feline WB samples (58, 59). The presence of PLT aggregates in the samples of this study could be related to the collection method, since the WB samples were obtained directly by catheterization of the cephalic vein using a 22 G catheter. It is well-known that the lesion of vascular endothelium produced by venipuncture causes PLT adherence to von Willebrand factor bound to subendothelial collagen with PLT GPIbα receptors, which induces additional PLT recruitment (60, 61). PLT counts by ADVIA were falsely decreased in samples with EDTA-induced pseudothrombocytopenia and was confirmed in the corresponding blood smears. Since pseudothrombocytopenia is a common occurrence in cats, the results of the samples with and without aggregates were included in the same statistical study, which could have induced the variations in PLT numbers in WB.

In this study, the MPV did not vary between the different analyzed samples (WB, PRP, and PPP). The results in relation to this parameter seemed controversial between studies. Indeed, compared to PLT concentrate, MPV increased in WB samples (33) although higher values in PLT concentrates compared to WB have been reported (27). MPV represents the average size of PLTs, increasing during PLT activation (62) and dependent on factors, such as anticoagulant, temperature and storage time of the blood sample (63–65). Although ADVIA is one of the best methods for PLT analysis (53), feline blood can contain PLTs larger than 60 fL (66) and may not be detected by the analyzer. This failure to detect large PLTs may be the reason why the number is falsely lower in some of the samples tested. Also, a low number of large PLT may not sufficiently increase MPV above the upper limit of the reference range.

Studies in cats with naturally occurring FeLV infections are valuable in providing practical clinical guidance and outcome expectations. The diagnosis carried out on these animals consisted of a rapid test (ELISA), detecting the presence of the virus (antigen) in blood, which does not always indicate the presence of the disease. Since these cats lived in an endemic area, the probability of false positives in the test could be excluded. As the test was repeated between 180 and 270 days after the first diagnosis of FeLV, these animals were considered to have the progressive form of the disease. Positive results could not be due to vaccination or maternal transfer of the virus, since the animals were adults and did not receive a vaccination regimen. The normal erytrogram and leukogram in the present study reinforce the hypothesis of the absence of BM involvement, since anemic, leukopenic, or thrombocytopenic animals were excluded. It is common that in persistently infected cats with the progressive form of infection hematopoietic cells invade the bone marrow (42), causing neoplastic hematopoietic pathologies, such as lymphoma (43), myelodysplastic syndrome (48), or non-neoplastic, including non-regenerative or regenerative anemia, immunosuppression, lymphopenia, neutropenia, and thrombocytopenia (35, 44–47). A leukogram characterized by leukocytosis with right shift neutrophilia and lymphopenia in response to stress was identified in two cats. Asymptomatic FeLV-positive animals are known to be less likely to have reduced blood counts than symptomatic ones (47). However, the type of viral strain, its pathogenicity and the type of study with experimentally vs. naturally infected animals may explain these discordant findings (67, 68), though future studies are needed to elucidate it.

Although there is no previous evidence on the effect of viral infections on the quality of PLT concentrate in cats, PDGF-BB and TGF-ß1 concentrations were determined in FeLV-positive animals according to the PRGF^®^-Endoret^®^ protocol previously described (33). In contrast to Silva's et al. (27) study, lower values of PDGF-BB were obtained in FeLV cats but similar or higher values of TGF-ß1 compared to healthy cats. In addition, in FeLV positive cats, lower values of PDGF-BB but similar values of TGF-ß1 than in healthy cats (33) have been obtained. Several reports in humans have documented elevated TGF-ß1 in blood, lymphoid tissues and cerebrospinal fluid in people infected with human immunodeficiency virus (HIV) (69). This elevation of cytokines was associated with defective T-cell recall, as well as B-lymphocyte proliferative responses and immunoglobulin production related to proapoptotic mechanisms (69). The marked increase in TGF-ß1 with advancing HIV-1 infection suggests an important immunosuppressive role of TGF-ß1 in the pathogenesis of this infection (70, 71). On the other hand, PDGF-BB were associated with severe disease in humans with COVID-19. This pro-inflammatory mediator indicates that innate immune cell responses and anti-viral T-cell responses are responsible for SARS-CoV-2 pathogenesis in COVID-19 patients (72).

The goal of this study was to characterize PRP and to determine GFs in both PRP and PPP fractions in FeLV positive cats. One of the most important limitations of this study was the small sample size in which only positive asymptomatic animals were considered, therefore, it cannot be considered representative of the entire FeLV positive feline population. The lack of a control group or the use of plasma from negative animals, same as the use of samples from positive animals in which the virus was purified by extraction with triton x 100 compared to the immunoassay for the detection of the FeLV p27 antigen used in our study has not allowed us to obtain comparative results.

On the other hand, since the GFs in PLT concentrates are modified by the type of PLT activator and other factors such as time and temperature, it would have been interesting to assess to what extent the time and temperature before proceeding to the freezing of the samples could have modified the concentrations of TGF-ß1 and PDGF-BB in this study. Taking these limitations into account, further research with regards to GFs obtained by PRGF^®^-Endoret^®^ methodology is needed. Since PRP obtained in our study showed similar characteristics to PRP products following PRGF^®^-Endoret^®^ system, asymptomatic animals could benefit from the use of GFs in cutaneous wounds, osteoarthritis, or bone fractures to improve their quality of life. since there is no cure for FeLV. However, further studies are needed to evaluate and define the potential clinical applications of PRP in FeVL-positive cats.

## 5. Conclusions

Using PRGF^®^-Endoret^®^ technology, it was possible to obtain and differentiate the PRP and PPP fractions in FeLV positive cats. The moderate PLT enrichment and the absolute reduction of RBCs and WBCs in the samples obtained have allowed the product to be classified as a P-PRP, although the minimum required for the PLT concentrate was not reached. The optimization and standardization of the protocol for the use of PRGF could represent an alternative to alleviate the side effects induced by the virus in feline patients.

## Data availability statement

The original contributions presented in the study are included in the article/supplementary material, further inquiries can be directed to the corresponding author.

## Ethics statement

The animal study was reviewed and approved by Animal Welfare Ethics Committee (CEEA) of the CEU Cardenal Herrera University in Valencia (Spain). Written informed consent was obtained from the owners for the participation of their animals in this study.

## Author contributions

LM-P performed the experiment. LM-P and KS analyzed data and wrote the manuscript and developed the first draft of the manuscript. DC, MT-T, and PP participated in performing the experiment. JMC, MR, and JS designed the study, supervised all procedures, and coordinated the research. MR and KS performed statistical analysis. JJC analyzed blood samples. JS proofread the manuscript and gave final approval of the version. All authors have read and agreed to the published version of the manuscript.
